# Large dynamic range pressure sensor based on two semicircle-holes microstructured fiber

**DOI:** 10.1038/s41598-017-18245-6

**Published:** 2018-01-08

**Authors:** Zhengyong Liu, Lin Htein, Kang-Kuen Lee, Kin-Tak Lau, Hwa-Yaw Tam

**Affiliations:** 1 Photonics research center, Department of Electrical Engineering, The Hong Kong Polytechnic University, Hung Hom, Kowloon Hong Kong; 2 Faculty of Science, Engineering and Technology, Swinburne University of Technology, John Street, Hawthorn, VIC 3122 Australia

## Abstract

This paper presents a sensitive and large dynamic range pressure sensor based on a novel birefringence microstructured optical fiber (MOF) deployed in a Sagnac interferometer configuration. The MOF has two large semicircle holes in the cladding and a rectangular strut with germanium-doped core in the center. The fiber structure permits surrounding pressure to induce large effective index difference between the two polarized modes. The calculated and measured group birefringence of the fiber are 1.49 × 10^−4^, 1.23 × 10^−4^, respectively, at the wavelength of 1550 nm. Experimental results shown that the pressure sensitivity of the sensor varied from 45,000 pm/MPa to 50,000 pm/MPa, and minimum detectable pressure of 80 Pa and dynamic range of better than 116 dB could be achieved with the novel fiber sensor. The proposed sensor could be used in harsh environment and is an ideal candidate for downhole applications where high pressure measurement at elevated temperature up to 250 °C is needed.

## Introduction

Microstructured optical fibers (MOFs) possess many advantages in sensing applications because of the flexibility in modifying their mechanical and optical properties, tailored for different applications. Sensors based on MOFs permit physical parameters such as strain^1^, pressure^2^, torsion^3^, as well as biomedical parameters like refractive index^4^, DNA^5^, etc., to be measured accurately. One of the most demanding applications is perhaps in downhole pressure measurement because it requires high-resolution and large dynamic range measurement in hostile conditions. Most commercially available pressure sensors are electrical transducers which generally can not operate at high pressure (>100 MPa) and high temperature (>200 °C)^6^. On the other hand, fiber optic sensors based on conventional optical fibers require bulky mechanical transducers to increase their sensitivity for pressure measurement^7,8^. However, MOFs for measuring pressure with high sensitivity as well as large measurement range can be realized. These features coupling with the capability of long distance measurement, make MOFs-based pressure sensors particularly promising in some applications, including downhole pressure measurements.

Optical fiber sensing technology based on fiber Bragg grating (FBG) inscribed in single mode fiber (SMF) and MOFs has been developed extensively for a wide range of applications. However, The typical pressure sensitivity of FBG written in SMF is only 3–4 pm/MPa^9^, too small for the discrimination of low pressure variation. Specially-designed optical fiber with air-hole structure along the fiber was fabricated to improve pressure sensitivity up to 20–30 pm/MPa^10–12^. This was a significant improvement but is still too small for many applications. The relatively insensitivity of these fibers to pressure is due to their low intrinsic modal dependence to pressure^13^. Owing to the ease of tailoring the microstructure patterns in MOFs, birefringence can be easily introduced in MOFs to realize highly sensitive pressure sensors using the Sagnac interferometer (SI) configuration. Birefringence in an optical fiber is introduced by the effective index difference between the two polarized modes, and SI-based pressure sensors employ the dependence of index difference with respect to pressure instead of only one modal effective index. SI-based sensor constructed with a polarization-maintaining photonic crystal fiber (PM-PCF) was demonstrated with high pressure sensitivity of 3,420 pm/MPa^2^. This is about 1000 times more sensitive compared with the FBG-based pressure sensors. Higher pressure sensitivity of ~6,850 pm/MPa^14^ was reported by using an MOF with a hole pattern arranged in a “butterfly” shape. Rocking filter was also fabricated in the “butterfly” MOF to realize pressure sensor. Ultra-high pressure sensitivity varying from ~81,000 pm/MPa to 177,000 pm/MPa was reported with the rocking filter-based sensor^15^. However, its sensitivity changed significantly with pressure and its use is limited to small measurement range. Apart from polarimetric sensors, MOFs-based pressure sensors employing other configurations such as Fabry-Pérot interferometer (FPI) was also reported but could only achieve sensitivity no more than 10 pm/MPa. For example, pressure sensitivity of ~6.78 pm/MPa was reported for a four-hole suspended-core fiber^16^, and ~5.77 pm/MPa was demonstrated using a solid-core photonic crystal fiber (PCF)^17^). It is worthy noting that FPIs employing thin diaphragms (e.g. silica diaphragm^18–21^, silver diaphragm^22^) show much higher sensitivity. Typically, the diaphragm deflection is used to estimate the pressure sensitivity (*S*_*D*_), sensitivity as high as 11,000 nm/MPa (i.e. spectral sensitivity^23^ (*S*_*λ*_)= $\lambda /{L}_{{cavity}}\bullet {S}_{D}$ = ~1,420 nm/MPa at 1550 nm) was reported by using silica film with ~12 µm cavity length^18^, and even higher sensitivity of 70,500 nm/MPa, (i.e. *S*_*λ*_ = ~376 nm/MPa) was achieved using thin silver diaphragm^22^. This type of miniature pressure sensors is commercially available from FISO Technologies Inc. and are designed for medical applications owing to the high sensitivity in small pressure range. However, such extrinsic sensors cannot sustain high pressure as diaphragm could be damaged and the performance highly depends on the sensor fabrication process and diaphragm materials. Higher damage threshold (e.g. >10 MPa) is possible, but with lower sensitivity^20^. Thus, pressure sensors with high measurement sensitivity and capable to work over a large operation range in harsh environment is highly desirable.

In this paper, we present a novel and simple MOF for pressure measurement with large dynamic range and high sensitivity. The in-house fabricated MOF contains two large semicircle holes in the cladding and a rectangular strut with core located in the center. The measured group birefringence of this fiber is 1.23 × 10^−4^, which is in good agreement with the simulation value of 1.49 × 10^−4^. Pressure sensor constructed with the novel MOF in a Sagnac interferometer configuration exhibited pressure sensitivity up to 50,000 pm/MPa, which is more than 10 times higher than any reported pressure sensors based in SI configuration. Pressure measurement was conducted up to 50 MPa, which was limited by the equipment available in our laboratory. However, optical fiber sensors based on fused silica have been demonstrated to withstand very high pressure and measured pressure up to 200 MPa was reported^12,24^. Such optical fiber sensor would need to be able to measure pressure as small as 1 kPa, if it was to achieve better than 106-dB dynamic range. For the proposed MOFs pressure sensor made of silica, spectral measurement accuracy of better than 50 pm is required. This is achievable as commercial spectral interrogators with 1-pm accuracy are readily available. Obviously, the minimum detectable pressure depends on the noise introduced by the spectral measurement system.

## Results

### Fiber fabrication

**Figure 1:**
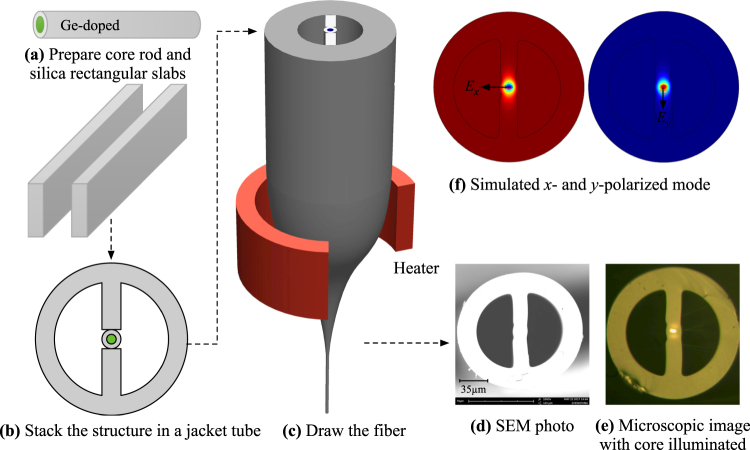
Schematic of the fabrication process of the two semicircle holes MOF (TSH-MOF) shown in (**a**–**c**); the cross-sectional photos of the fabricated TSH-MOF taken by (**d**) scanning electron microscope (SEM) and (**e**) microscope with core illuminated. (**f**) shows the simulated electric field distribution of the two modes polarized orthogonally in *x* and *y* direction.

Figure 1 illustrates the fabrication process of the two semicircle holes MOF (TSH-MOF). Firstly, a core rod doped with germanium and two rectangular silica slabs are prepared. The stack-and-draw approach is employed to make the fiber. During the stacking processing, the two rectangular sheets were placed in a straight line inside a jacket tube with the core rod fixed in the middle between these two silica slabs, as shown in Fig. 1(b). The fiber was drawn from the stacked preform on a fiber draw tower at a drawing temperature of ~1910 °C. Figure 1(d) shows the scanning electron microscopic (SEM) photo of the cross section of the fabricated fiber. The two large semicircle holes in the preform are maintained in the drawn fiber. The fiber has a cladding diameter of ~125 µm and an elliptical core with its major and minor axis of ~6 × 3 µm, as shown in Fig. 1(e). The semicircle hole has a radius of ~40 µm. In contrast to other types of side hole fiber where the long axis of elliptical core is parallel to the fiber symmetry axis^25^, the long axis of the elliptical core of the TSH-MOF is perpendicular to the fiber symmetry axis. This enhances the stress difference distributed along two polarized axes under pressure, resulting in increased sensitivity. Propagation loss of the fabricated fiber measured by cut-back method is ~0.5 dB/m, which is low enough for sensing applications. The TSH-MOF was manually spliced to conventional SMF, to reduce hole collapse. The average splicing loss is ~0.5 dB/splice and is very easy to achieve. The relatively high splicing loss compared with SMF-to-SMF splicing is due to the mode mismatch and large air holes. Figure 1(f) shows the electric field distribution of the two supported polarized modes, corresponding to *E*_*x*_ and *E*_*y*_.

### Fiber birefringence and simulation results

**Figure 2:**
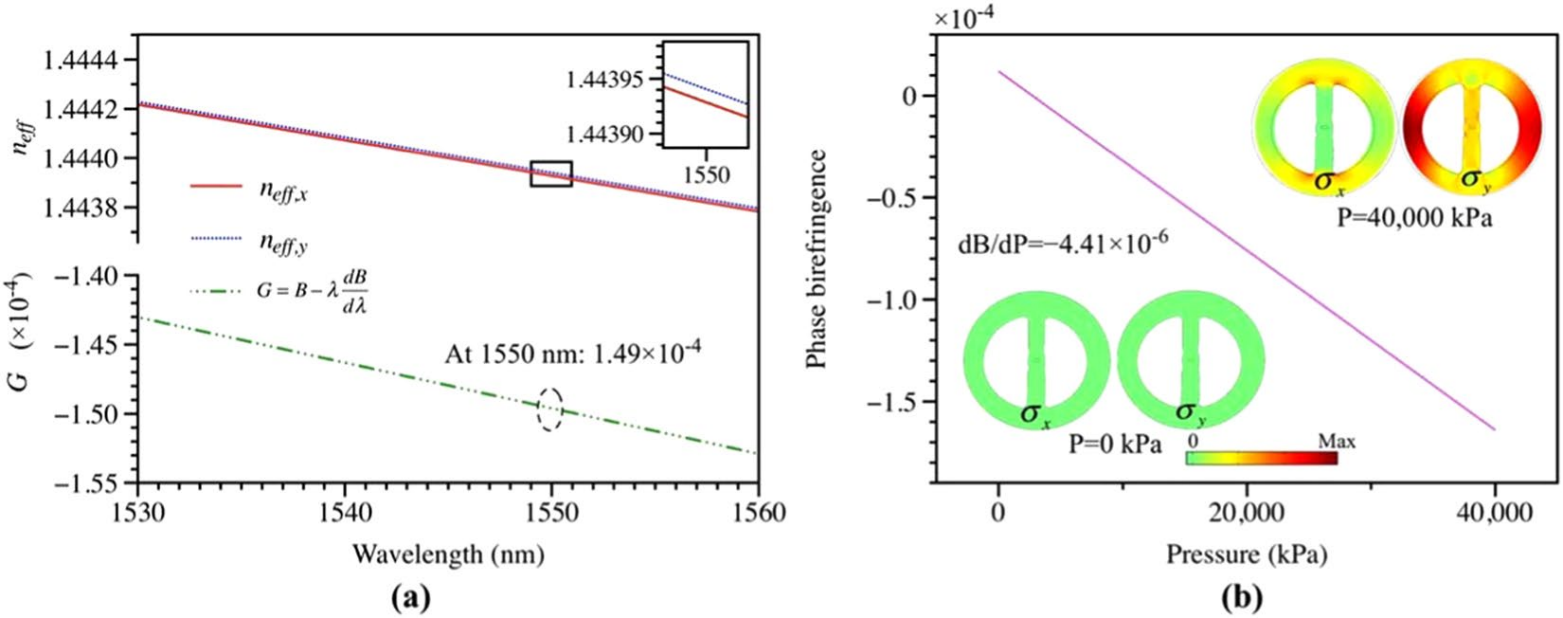
(**a**) Calculated effective index of the two polarized modes and group modal birefringence with respect to wavelength, and (**b**) calculated linear response of phase modal birefringence with applied pressure on the outer boundary of the fiber. Insets of (**b**) show the stress distribution on *x* and *y* direction under the applied pressure of 0 kPa and 40,000 kPa.

The TSH-MOF is birefringent, and FEM simulation is performed to investigate its birefringence and polarimetric pressure sensitivity using the actual fiber cross-section obtained from the SEM photo. The refractive index of silica is calculated with the Sellmeier equation using the value of 0.006 as the index difference between core and silica. According to the simulation results, the fabricated TSH-MOF supports two orthogonal polarized modes (shown in Fig. 1(e)), which has effective index of 1.44392852, 1.44394063 for *x-* and *y-*polarized modes, respectively. This means the fast axis is along the *x* polarization. The effective index of both polarized modes decreases with wavelength, as shown in Fig. 2(a). As a result, the group modal birefringence (*G*) can be obtained via the relationship $G=B-\lambda \frac{{dB}}{d\lambda }$, where *B* represents the phase modal birefringence and equals the effective index difference of the two polarized modes. To calculate d*B*/d*λ*, the discrete values of $B({\lambda })={n}_{y}({\lambda })-{n}_{x}({\lambda })$, are fitted with a six-degree polynomial^26^. Figure 2(a) also plots the calculated group modal birefringence with respect to wavelength. The sigh of *G* is negative, meaning that anomalous chromatic dispersion of *B* is large, which is similar to the birefringent MOFs reported in literatures^14,26–28^. The value of *G* increases with wavelength, and is 1.49 × 10^−4^ at the wavelength of 1550 nm.

The two semicircle holes enhanced the induced stress asymmetrically in the fiber core when the fiber is subjected to pressure, resulting in increased pressure measurement sensitivity. The transmission spectrum of a TSH-MOF pressure sensor based on SI configuration can be approximated as

1 $$T=\frac{1-\,\cos (\delta )}{2},$$

where $\delta$ is the phase difference induced by the TSH-MOF in the Sagnap loop and equals to (2π/λ)*BL*, *L* is the length of the fiber. As the phase difference is always equal to 2kπ (k ∈ I) at the minima position of the spectrum, the pressure sensitivity of the sensor in terms of wavelength shift can be derived as

2 $$\frac{d\lambda }{dP}=\frac{\lambda }{G}\frac{dB}{dP}.$$

Basically, when the fiber is subjected to pressure, the stress distribution induced by the applied pressure is not uniform due to the existence of asymmetrical air-hole structure. The pressure-induced change in the refractive index of the MOF due to photoelastic effect can be expressed by

3 $$[\begin{array}{c}\begin{array}{c}{\rm{\Delta }}{n}_{x}\\ {\rm{\Delta }}{n}_{y}\\ {\rm{\Delta }}{n}_{z}\end{array}\end{array}]=[\begin{array}{ccc}{C}_{1} & {C}_{2} & {C}_{2}\\ {C}_{2} & {C}_{1} & {C}_{2}\\ {C}_{2} & {C}_{2} & {C}_{1}\end{array}]\,[\begin{array}{c}\begin{array}{c}{\sigma }_{1}\\ {\sigma }_{2}\\ {\sigma }_{3}\end{array}\end{array}],$$

where Δ*n*_*i*_ (*i* represents *x*, *y*, *z*) is the index change of the stressed fiber; *σ*_1_, *σ*_2_, *σ*_3_ are the principal components of the pressure-induced stress; *C*_1_, *C*_2_ are the stress-optic coefficients and have values of −0.65 × 10^−12^ m^2^/N, −4.2 × 10^−12^ m^2^/N, respectively^29^. In the simulation, we assume the principle stress axes of the fiber and the Cartesian axes (*x*, *y*, *z*) overlap, which is adopted for anisotropic fiber^26^. Figure 2(b) plots the calculated phase modal birefringence with increasing pressure applied on the outer boundary, producing a response of d*B*/d*P* = −4.41 × 10^−6^. Taking the group modal birefringence of 1.49 × 10^−4^ into account, the pressure sensitivity of the pressure sensor using the TSH-MOF in a SI configuration is calculated to be 45,876 pm/MPa at the wavelength of 1550 nm, according to equation (2).

### Pressure measurement results

**Figure 3:**
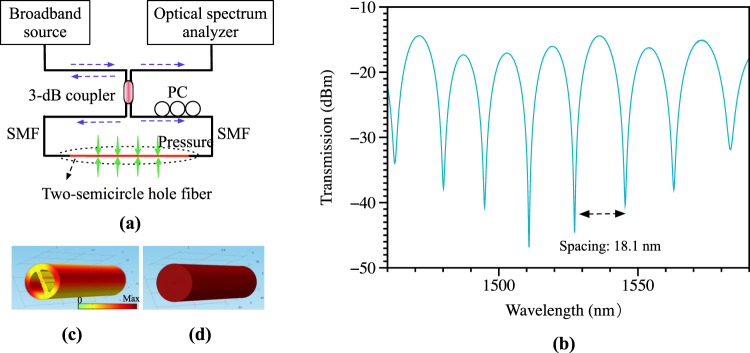
(**a**) Schematic figure of the Sagnac interferometer (SI) setup, and (**b**) the SI transmission spectrum using 1.058 m of the TSH-MOF; (**c**) and (**d**) show the stress distribution of the TSH-MOF and single mode fiber, respectively, under hydrostatic pressure of 40 MPa. The comparison clearly indicates the asymmetrical stress transferred by the semicircle holes to the fiber core when the fiber is subjected to pressure.

A 3-dB coupler was utilized to connect the TSH-MOF on one side into a Sagnac loop, as shown in Fig. 3(a). The coupler splits the input broadband light into two counter-propagating beams that interfere constructively or destructively at the coupler. The interference spectra were measured by the optical spectrum analyzer. The polarization controller (PC) inside the loop is used to adjust the polarization to achieve good extinction ratio in the spectrum. However, in actual application, polarization-maintaining fiber and polarization-maintaining coupler could be used instead of the PC for the realization of compact sensor. The reflection spectrum was also measured using an FBG-interrogator (sm125 from Micron Optics). The group modal birefringence of the TSH-MOF used in the SI can be written as

4 $$G=\frac{{\lambda }^{2}}{{\rm{\Delta }}\lambda \cdot L}\approx \frac{{\lambda }_{1}\cdot {\lambda }_{2}}{|{\lambda }_{1}-{\lambda }_{2}|\cdot L},$$

where *L* is the length of the TSH-MOF, *λ*_1_ and *λ*_2_ are two adjacent wavelength valleys in the spectrum, and Δ*λ* represents their spacing. Figure 3(b) plots the transmission spectrum obtained using a fiber length of 1.058 m, showing a separation of ~18.1 nm around the wavelength range of ~1550 nm. Thus, the value of *G* measured in the experiment is ~1.23 × 10^−4^, which is in good agreement with the simulation result.

Owing to the existence of the two semicircular air holes, the stress distribution induced by the external pressure is asymmetric, which differs from the stress transfer in the case of conventional single mode fiber, as shown in Fig. 3(c) and (d). The two semicircle holes totally cut off the stress transferred to the core along the fast axis. Based on photo-elastic effect, such distribution leads to large pressure-induced change in birefringence (i.e. d*B*/d*P*). Furthermore, the major axis of the elliptical core is perpendicular to the central bridge, thus induces relatively small value of group modal birefringence (i.e. *G*) and enhances the pressure sensitivity.

**Figure 4:**
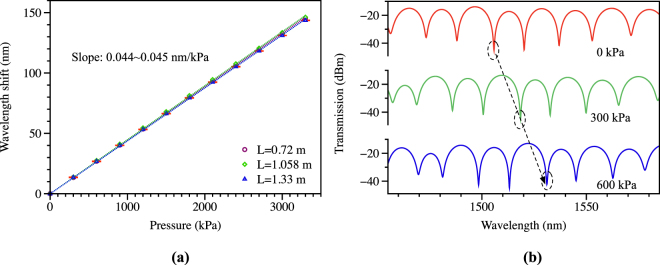
Pressure measurement results: (**a**) wavelength shift of one valley as a function of applied pressure for the 0.72 m long TSH-MOF (circle points), 1.058 m (square points), and 1.33 m (triangle points), producing sensitivity of 44~45 pm/kPa; (**b**) spectra for the case of 1.058 m long TSH-MOF at 0 kPa, 300 kPa, and 600 kPa.

To confirm the high sensitivity, we conducted pressure measurements by using the SI configuration shown in Fig. 3(a), where the TSH-MOF is totally immersed in a hydrostatic oil pressure environment. The experimental results are plotted in Fig. 4, where (a) shows the measured wavelength shift of one valley with increasing pressure for fiber lengths of 0.72, 1.058, and 1.33 m, and (b) is the corresponding spectra plotted for the case of 1.058 m under pressure of 0, 300, and 600 kPa. Even though various fiber lengths were utilized, all the measurement results produced similar sensitivity of 44–45 nm/MPa. As expected, the contribution of the fiber length to pressure sensitivity can be neglected, as assumed in equation (2). Furthermore, the measured pressure sensitivity based on the TSH-MOF is in good agreement with the calculated result of 45.876 nm/MPa. The length independent feature makes it possible to produce compact sensors suited for applications with limited space. As indicated in Eq. (4), longer fiber length decreases the spectral fringe spacing and results in sharper wavelength dip, thus allowing more accurate measurements. In practical application, there is a tradeoff between the fiber length and measurement accuracy. Nevertheless, the fiber can be coiled into a compact sensor as the bending curvature does not affect its sensitivity.

**Figure 5:**
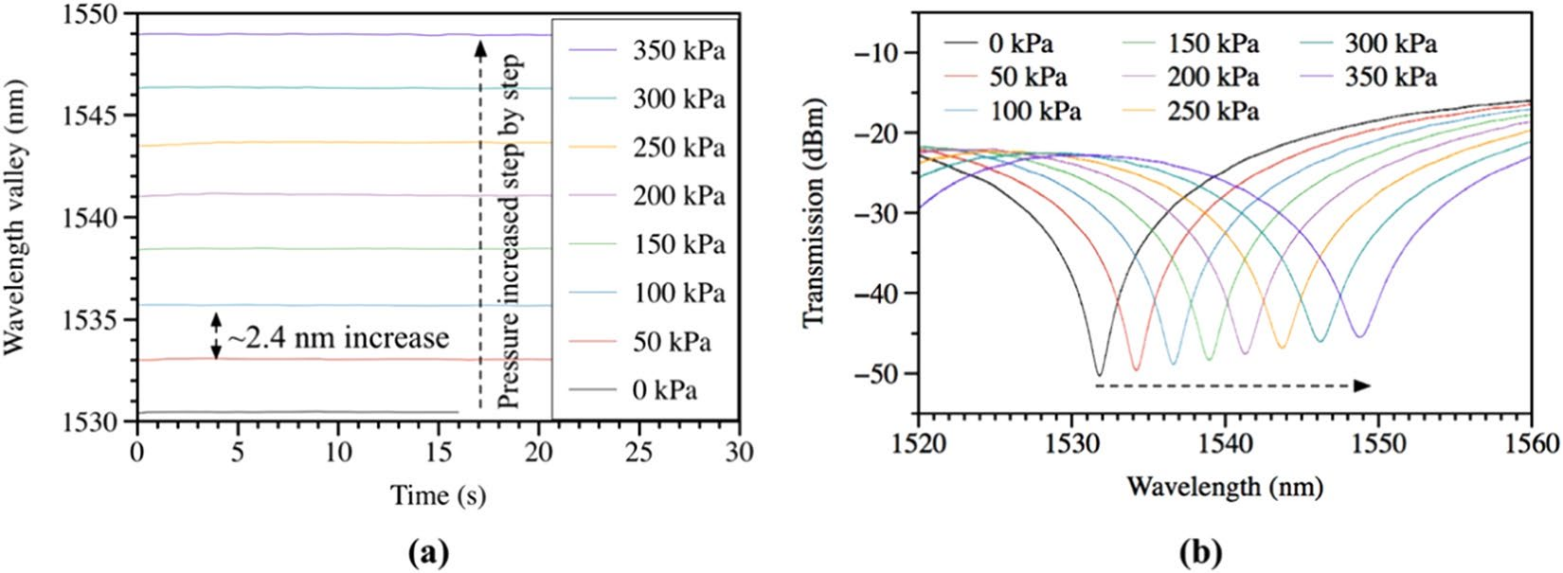
Experimental results of low-pressure measurement up to 350 kPa with step of 50 kPa: (**a**) wavelength change with pressure and (**b**) the spectra shift of a valley.

In addition to the high-pressure measurement experiment, low-pressure measurement experiments were also conducted for the two pressure ranges of 0–800 kPa, and 0–45 kPa with steps of 50 kPa and 3 kPa, respectively. Figure 5 shows the experimental results of one valley’s wavelength change (a) as well as spectra shift (b) with pressure up to 350 kPa. Figure 5(a) shows that every 50-kPa of pressure induces a large wavelength shift of 2.4 nm. All the spectra shown in Fig. 5(b) are very clean and the wavelength dips are more than 20 dB from the transmission peaks, allowing accurate measurement of wavelength shift.

**Figure 6:**
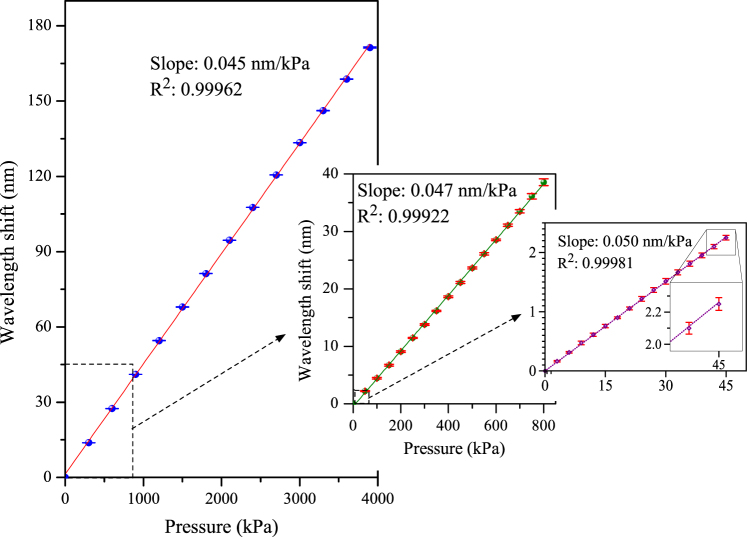
Pressure responses of the TSH-MOF sensor for the measurement ranges of: 0–4000 kPa, 0–800 kPa, 0–45 kPa.

The sensitivities in the pressure ranges of 0–800 kPa, and 0–45 kPa were measured to be ~0.047 nm/kPa, and 0.050 nm/kPa, respectively. Figure 6 shows the plots of the wavelength shift as a function of the applied pressure, where the insets show the results of the small-pressure experiments that were conducted separately. The increase in sensitivity in the smaller pressure range is due to the change of group modal birefringence with pressure. All the measurements were repeated three times and very small errors (less than ±40 pm as shown in the inset) were observed even for small pressure change of 3 kPa. The measurement accuracy is estimated to be ±1 kPa.

### Sensitivity and dynamic range

**Figure 7:**
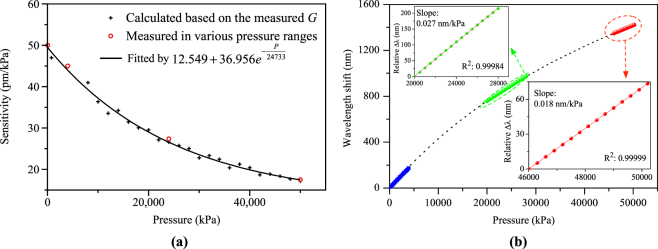
(**a**) Measured and calculated pressure sensitivities with respect to applied pressure; and (**b**) calculated wavelength shift (dashed line) as function of the full range pressure. Insets show the measured responses for the pressure ranges of 20,000–28,000 kPa and 46,000–50,000 kPa.

According to Eq. (2), the pressure sensitivity is related to the group birefringence. The group birefringence of the TSH-MOF at different pressures is measured experimentally from the Sagnac interference spectrum and calculated using Eq. (4). The group birefringence increases slightly with applied pressure due to slight deformation of the large two semicircle holes in the cladding. The pressure sensitivity can be calculated using Eq. (2), which is plotted in Fig. 7(a). The measured sensitivities in various pressure ranges are also plotted, showing good agreement with the calculated results. Figure 7(b) plots the calculated wavelength shift from 0 to 50,000 kPa by conducting the integral of the fitted equation of sensitivity with respect to pressure. The measured relative wavelength shifts in the ranges of 20,000–28,000 kPa and 46,000–50,000 kPa are shown in the insets of Fig. 7(b). The experimental results are in excellent agreement with the calculated results. The highest applied pressure is 50,000 kPa, which is limited by our test rig. However, silica based fiber has been demonstrated to withstand over 200,000 kPa^24^ of pressure. The sensitivity at 200,000 kPa is estimated to ~0.013 nm/kPa according to the fitting equation, which is also more sensitive than other reported MOFs.

**Figure 8:**
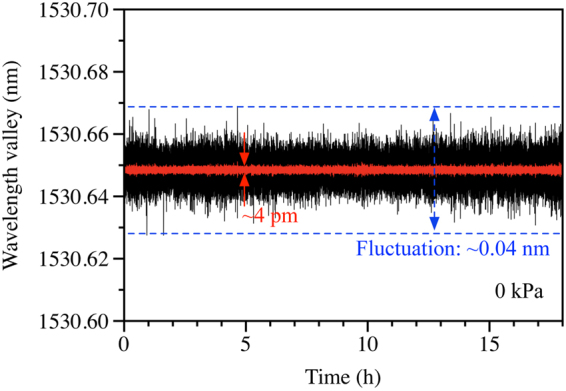
Measured fluctuation of one valley in the interference spectrum for over 18 hours when the applied pressure is 0 kPa. The black plot shows the data acquired at 2 Hz and the red plot is the 10-points averaging of the same set of data.

The pressure sensor constructed with the TSH-MOF in a Sagnac configuration was connected to an FBG-interrogator (sm125 from Micron Optics) which has a 1-pm wavelength measurement accuracy and 0.2-pm resolution. The wavelength dip of one valley of the spectrum was measured continuously over 18 hours at a sampling rate of 2 samples/sec to show the system noise level over a relatively long-period, when the sensor was subjected to 0 kPa. Figure 8 shows the measured data and the peak-to-peak fluctuation is around 40 pm. The small-pressure sensitivity of the sensor is 50 pm/kPa (refer inset of Fig. 6), giving a pressure measurement variation of about 0.6 kPa. Applying 10-point averaging to the same set of data significantly reduced the fluctuation to about 4 pm, shown in red in Fig. 8. This corresponds to pressure measurement variation of 0.08 kPa, which indicates that the minimum detectable pressure of 0.08 kPa can be obtained by the proposed sensor.

The dynamic range (*DR*) of the pressure sensor is determined by the largest and lowest pressures measured by the sensor. Figure 6 shows the maximum pressure measured by the sensor is 50,000 kPa. However, sensors based on silica optical fibers can withstand extremely high pressure. Pressure measurement with silica fiber sensor beyond 200 MPa was reported^24^. The smallest detectable pressure is limited by the measurement accuracy, spectral stability (i.e. noise) as well as the sensor resolution. The result shown in Fig. 8 shows that the minimum detectable wavelength fluctuation is 4 pm, which corresponds to minimum detectable pressure of 0.08 kPa by the TSH-MOF sensor. The dynamic range of the sensor can be calculated by

5 $$DR(dB)=20\times {\mathrm{log}}_{10}(\frac{{P}_{\max }}{{P}_{\min }}),$$

where *P*_max_, and *P*_min_ are the maximum and minimum pressures that the sensor can measure. Larger *DR* means wider measurement range. The DR of the TSH-MOF-based SI pressure sensor obtained experimentally is 115.9 dB and could be higher than 128 dB, if pressure measurement facility of up to 200 MPa was available.

It is worth noting that pressure sensors based on FPIs constructed by depositing thin films (e.g. silver diaphram^22^) close to the fiber facet exhibit very high sensitivity due to the nanometer thick film. However, this kind of sensors may not be able to withstand high pressure and has small dynamic range.

## Discussion

**Figure 9:**
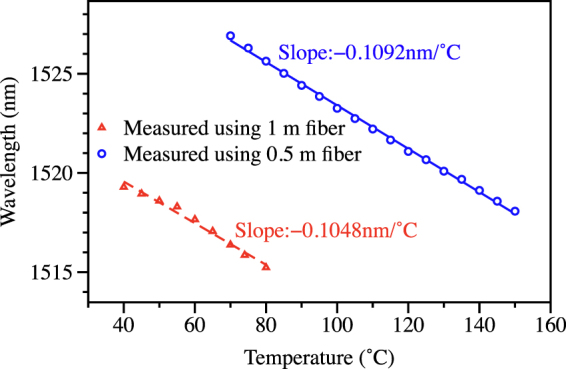
Experimentally measured temperature dependence of the TSH-MOF sensor using fiber length of 1 m and 0.5 m, showing a temperature response of −105 pm/°C and −109 pm/°C, respectively.

The proposed pressure sensor constructed with a novel two semicircle-holes microstructure optical fiber exhibits excellent performance in terms of sensitivity and dynamic range. Figure 9 plots the temperature responses of the sensor with fiber length of 1 m and 0.5 m, respectively. The measured temperature dependence is about ~100 pm/°C, meaning that the temperature cross-sensitivity is about 2 kPa/°C. The temperature effect is not negligible but could be compensated easily with a fiber Bragg grating (FBG) inscribed in the photosensitive germanium-doped core of the TSH-MOF. FBG typically has a temperature coefficient of 10 pm/°C and can measure temperature with an accuracy and resolution of better than 0.1 °C, and 0.02 °C, respectively by using the sm125 interrogator.

The spectra of the FBG and the TSH-MOF sensors measured by an FBG-interrogator contain the pressure and temperature information. Therefore, simultaneous measurement of pressure and temperature can be realized using the TSH-MOF. It is an intrinsic optical fiber sensor coated with polyimide and thus can operate at high temperature beyond 200 °C. These features make the sensor particularly suited for harsh environment applications such as monitoring of high pressure and high temperature in downholes. One of the leading products in the market for downhole measurement/monitoring is the Signature CQG crystal quartz gauge developed by Schlumberger^30^. Table 1 shows the specifications of the CQG crystal quartz gauge pressure and temperature sensor, and also the corresponding performance of the proposed TSH-MOF sensor. The preliminary results of the TSH-MOF sensor show that most of the specifications of the novel microstructured optical fiber sensor are superior to the commercial crystal quartz gauge sensors.

**Table 1:** Performance comparison between a leading market product and the proposed TSH-MOF sensor for downhole measurements.

Parameters	CQG Crystal Quartz Gauge	TSH-MOF SI Sensor
Pressure rating, MPa	110	>200
Temperature rating, degC	175	250
Pressure Accuracy, kPa	±8.3	better than 1
Pressure Resolution, kPa	0.021	0.015*
Temperature Accuracy, degC	±0.2	0.1
Temperature resolution, degC	0.001	0.02
Scanning rate	0.1 s to 10 min	0.001 s** to 0.5 s

Note: The temperature specifications are for typical germanium-doped fused silica optical fibers. *Estimated based on interrogator resolution of 0.2-pm and pressure sensitivity at 200 MPa; **1000 Hz scanning rate FBG-interrogator with 1-pm accuracy.

In conclusion, we reported a novel high-birefringence microstructured optical fiber, consisting of two large semicircle holes in the cladding, for measurement of pressure in harsh environment with high sensitivity and very large measurement range. Both simulation and experiment were carried out to verify and demonstrate the pressure sensitivity based on Sagnac interferometer configuration. The calculated group modal birefringence of the fabricated fiber is 1.49 × 10^−4^ at the wavelength of 1550 nm, which agrees well with the value measured in the experiment (1.23 × 10^−4^). Repeatable pressure tests confirm the high sensitivity, giving measured values of 45 nm/MPa in the range of 0–4,000 kPa, 47 nm/MPa in the range of 0–800 kPa, and 50 nm/MPa in the range of 0–45 kPa. The capability of the fiber optics sensor to measure pressure from 80 Pa to more than 200 MPa, demonstrated that the sensor is an ideal candidate for downhole pressure measurement.

## Methods

### Fiber fabrication

To fabricate the two semicircle holes MOF, a germanium doped core rod with core and cladding diameter of around 0.7 × 0.3 mm and 2.5 mm was used. This rod was drawn after milling a preform having core and cladding diameter of 4 mm and 22.5 mm in rectangular shape. The index difference between core and cladding is ~0.006. Two rectangular silica slabs that have thickness of ~2.5 mm and width of ~8 mm were utilized to stack the structure in a jacket silica tube with inner and outer diameter of 19 mm and 25 mm, respectively. Careful alignment was conducted to ensure the core rod was placed in the center position of the tube, at the same time fixed tightly by the two rectangular slabs. The stacked preform was drawn into abovementioned TSH-MOF on the fiber draw tower at a temperature of about 1910 °C. Relatively lower drawing temperature was employed in order to preserve the semicircle hole structure, especially with such large hole size. The feeding and drawing speed were set to be 0.3 mm/min and ~10 m/min, respectively. Apart from low drawing temperature, the drawing tension was maintained between 0.7 and 0.8 N, which is relatively high as well to keep the large air hole structure. The final fiber was coated by polyimide.

### Fiber connection with SMF

To connect the fabricated TSH-MOF with SMF, manual splicing process was carried out to reduce the loss to acceptable level, similar to our previous reported MOFs^31^. The splicing process is very simple by using a commercial fuse splicer (FITEL-S178). Except arc duration time and power, which were set to be 200 ms and 50 units, all the other parameters were disabled. Since the outer diameter of TSH-MOF is ~125 µm, which is matched with SMF, manual alignment was conducted according to the cladding boundary of both fibers. Generally, about 50 µm offset relative to the central arc position was introduced for SMF during manual alignment, which reduced the arc strength on TSH-MOF side to avoid possible collapse of air holes. Typically, about 7 times of arc discharge were sufficient to achieve good connection strength. Such approach only introduced a splicing loss of ~0.5 dB per one joint, which is acceptable for the sensing applications.
